# Emerging PFAS: Current Concerns, Remediation, and Future Perspectives

**DOI:** 10.3390/toxics14090792

**Published:** 2026-09-07

**Authors:** Ryuichi Mashima

**Affiliations:** Department of Clinical Laboratory Medicine, National Center for Child Health and Development, 2-10-1 Okura, Setagaya-ku, Tokyo 157-8535, Japan; mashima-r@ncchd.go.jp; Fax: +81-3-3417-2238

**Keywords:** emerging PFAS, remediation, biological transformation, ultrashort-chain PFAS, regulation

## Abstract

Emerging per- and polyfluoroalkyl substances (PFAS) constitute the most currently available PFAS used across many applications, such as aqueous film-forming foam, textiles, food contact materials, chromium plating, semiconductor production, and fluoropolymer producing aids. Since the Stockholm convention, legacy PFAS has been successfully replaced to emerging PFAS. In addition, emerging PFAS can be generated from industrial mitigation process of legacy PFAS, inadvertently from previously emitted fluorocarbons by photooxidation, and is generated for new application such as lithium battery electrolyte. This review first defines emerging PFAS in this manuscript followed by overviewing of their production and mitigation process based on recent advances in technology.

## 1. Introduction

**Figure 1:**
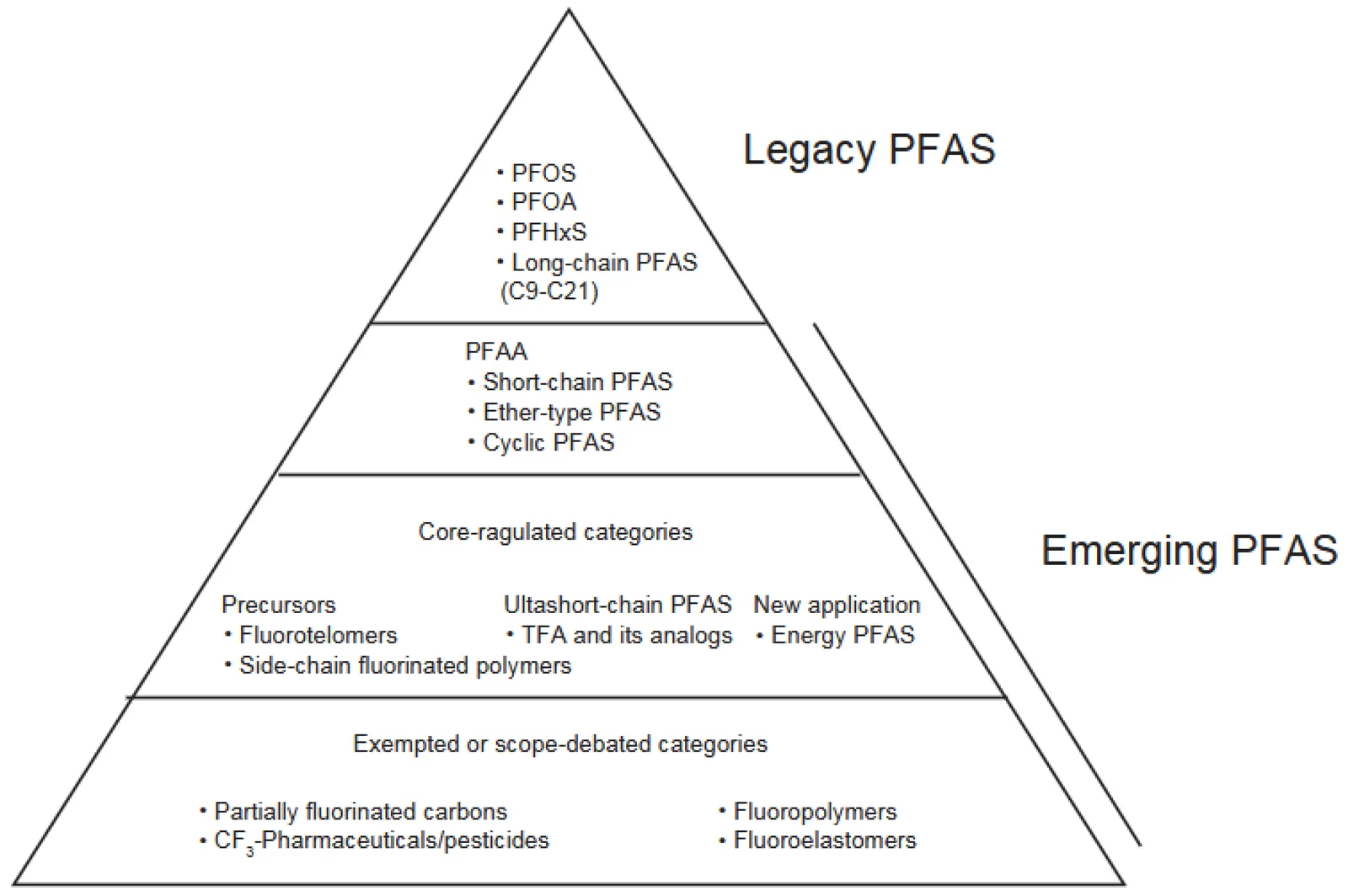
Legacy PFAS and emerging PFAS. PFAA—perfluorinatedalkyl acids.

Per- and polyfluoroalkyl substances (PFAS) are a group of fluorinated compounds that were first discovered more than seven decades ago [1,2] (Figure 1). PFAS have been used for aqueous film-forming foam [3,4,5], textiles [6,7,8], food contact materials [6,9,10,11], chromium plating [12,13,14], semiconductor production [15], and fluoropolymer processing aids [16,17,18] (Table 1). The chemical properties of PFAS include high chemical stability, high temperature stability, oil and water resistance, surfactant properties, and electrical insulation properties. These unique characteristics arise from the higher bond dissociation energy of C-F than C-H. Thus, PFAS are occasionally called “forever chemicals.” In 2021, the Organization for Economic Cooperation and Development (OECD) re-defined as “PFASs are defined as fluorinated substances that contain at least one fully fluorinated methyl or methylene carbon atom (without any H/Cl/Br/I atom attached to it), i.e., with a few noted exceptions, any chemical with at least a perfluorinated methyl group (–CF_3_) or a perfluorinated methylene group (–CF_2_–) is a PFAS” [1].

**Figure 2:**
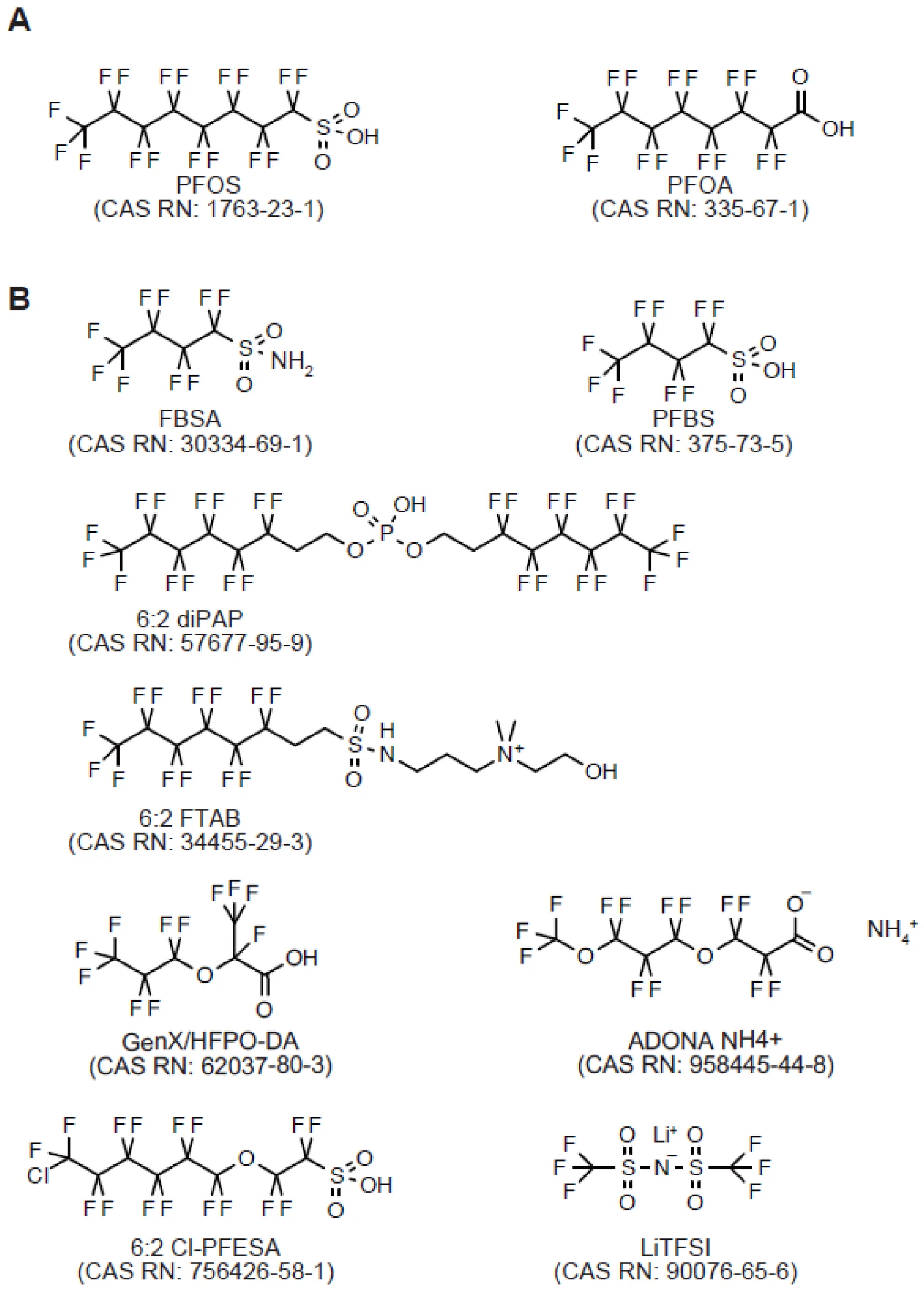
Some legacy PFAS and emerging PFAS. (**A**) Chemical structure of PFOS and PFOA. (**B**) Chemical structure of some emerging PFAS.

Under the Stockholm Convention, perfluorooctanesulfonate (PFOS), perfluorooctanoic acid (PFOA), and perfluorohexanesulfonate (PFHxS) were targeted for ban. In this review, the author defines the term ‘legacy PFAS’ as these restricted PFAS, longer PFAS, and their precursors (Figure 2) [1,22,23]. A precursor is defined as any chemical that readily gives PFAS. These compounds were previously synthesized but are now phased out (Figure 2) [1]. Precursors include sulfonamide derivatives, such as perfluorooctanesulfonamide (PFOSA); sulfonamide alcohol derivatives, such as *N*-methylperfluorooctanesulfonamidoethanol (*N*-MeFOSE); and PFOS phosphoesters, such as perfluorooctane sulfonamidoethanol-based phosphate (SAmPAP). 8:2 fluorotelomer alcohol (8:2 FTOH) esterified polyacrylate is another example of legacy PFAS because 8:2 FTOH can be converted into PFOA.

Emerging PFAS is newly developed compounds that do not include legacy PFAS [24,25,26]. Prototypical examples include perfluoroalkyl acids (PFAA), which include short-chain PFAS, ether-type PFAS, and cyclic PFAS [27]. In addition, newly recognized PFAS, which is not included in Buck et al. (2011) [27], includes following chemicals: (a) a chemical which only contains an isolated CF_3_-group such as trifluoroacetic acid (TFA), trifluoromethanesulfonate (TFMS), CF_3_-pharmaceuticals/pesticides; (b) a chemical with any only non-terminal CF_2_ lacking terminal CF_3_ (i.e., perfluorosuccinic acid); (c) a chemical with perfluorinated alkylated aromatic structure (i.e., 4-(3,3,4,4,5,5,6,6,7,7,8,8,8-tridecafluorooctyl)phenyl]methanol). Current concerns involve exempted or scope-debated PFAS categories, including partially fluorinated carbons, CF_3_-pharmaceuticals/pesticides, fluorinated electrolytes, fluoropolymers, and fluoroelastomers. In Buck et al. (2011), polymeric PFAS include (a) fluoropolymers, (b) side-chain fluorinated polymers (SCFP), (c) perfluoropolyethers [27]. Esterified fluoropolymers can easily release monomers such as fluorotelomers; thus, these can be considered precursors. From a regulatory point of view, in United States Environmental Protection Agency (EPA) documents, ‘emerging PFAS’ appears in a historical context and is not defined as any specific compounds. Because of this ambiguity, the European Chemicals Agency (ECHA) proposes broader regulation of these emerging PFAS, which has sparked discussion between regulatory authorities and industry (Figure 1).

In terms of global production, since the invention of PFOS in 1949, production increased and plateaued at 5000 tons during 1970 to 1990, then was phased out in 2010 [28]. In sharp contrast, the emerging PFAS were not seen in 2000. Since then, its production increased to 5,000 tons in 2010 and 35,000 tons in 2020 [29]. Based on these data, the production of emerging PFAS shows surprising expansion. This means that, apparently, PFAS has a variety of useful properties for humans regardless of age. More precisely, emerging PFAS production in 2010 was comparable to legacy PFAS production in 1980–2000, indicating that replacement from legacy PFAS to emerging PFAS occurred successfully. The further parabolic increase in emerging PFAS suggests that new industrial applications include lithium-ion batteries in the auto/aerospace industry [30,31] and proton exchange membrane water electrolyzers in power plants, semiconductor factories, and numerous on-site hydrogen refueling stations [32].

In this narrative review, the author examined the current status of emerging PFAS usage, industrial remediation methods, the development of PFAS alternative materials, and the current aim of proposing regulation. The cited literature was searched on PubMed and was mostly published in 2022–2026. A keyword search was performed using a combination of “emerging PFAS” AND another keyword. When necessary, Claude.ai was used for reference searches not found in PubMed. In this case, the reference information was further confirmed online.

## 2. Major Emerging PFAS Compounds Categorized by Functional Group

### 2.1. Short-Chain PFAS

Short-chain PFAS can be synthesized through either the electrochemical fluorination method or the fluorotelomer method [1,33]. The nomenclature of chemicals synthesized by fluorotelomer-based compounds begins with FT, the abbreviation for fluorotelomer. By contrast, the nomenclature of electrochemical fluorination appears confusing because the abbreviation of most compounds produced using this method begins with either F or P for fluoro- or perfluoro-, based on the name of the International Union of Pure and Applied Chemistry.

#### 2.1.1. Electrochemical Fluorination (ECF)-Derived Compounds

ECF method uses corresponding hydrocarbons as starting materials [34]. The reaction occurs at low voltage for several weeks to months at ambient temperature in hydrogen fluoride as a solvent. The reaction mechanism is believed to involve a mixture of radical and ionic pathways [34]. After the reaction completes, the resulting products are neutralized. The Major side products include branched compounds such as 6-perfluoromethylheptane sulfonate through a tricyclic intermediate. In addition, ECF-mediated PFOS production also generates both C6- and C5-cyclic sulfonates. This is attributed to the radical stabilization of cyclic intermediates.

##### Sulfonate

Sulfonate is often found in emerging PFAS [1]. Normally, the aqueous solubility of sulfonate PFAS compounds is higher than that of the corresponding carboxylic acid, particularly in long-chain PFAS. Therefore, C6 PFAS are used for aqueous film-forming foam and chromium plating. For chromium plating, adding these PFAS increases the surface force of the chromium plating agents, preventing chromium loss by evaporation at high temperatures. Sulfonate-containing C4 PFAS were initially used for semiconductor production after photolithography.

##### Sulfonamide

Perfluoroalkane sulfonamides and their derivative esters consist of a distinct class of emerging PFAS with high volatility [20,35]. Because of this property, sulfonamides are used in semiconductor production. Most C4 sulfonamide emerging PFAS are produced using electrochemical fluorination. Because a variety of esterifications can be performed, many sulfonamide derivatives exist depending on the application. Some sulfonamides are readily absorbed by bacteria and are susceptible to microbial biotransformation [9]. In this reaction, sulfonamide is normally transformed into a carboxylic acid. C4 PFAS are best used for etching solutions and subsequent washing solutions in semiconductor production.

#### 2.1.2. Fluorotelomer (FT)-Derived Compounds

The FT method proceeds via radical reaction [34,36]. For the production of FT-derived PFAS, in liquefied CF_3_CF_2_I, gaseous trifluoroethylene (CF_2_=CF_2_) is introduced to form a mixture of even-numbered fluoroalkyl iodides. After reaction with ethylene, the resulting FT iodide was converted to FTOH. Oxidation to carboxylic acid was performed by the conventional method. To produce a sulfonate, fluorotelomer iodide is converted to a sulfonate via sulfonyl chloride.

##### Carboxylic Acid

Carboxylic acid is another widely found functional group in emerging PFAS [1]. The acidity of carboxylic acid is weaker than that of sulfonate. Thus, the solubility of carboxylic acid PFAS is lower than that of sulfonate PFAS. This chemical property has been successfully used in the coating of textiles. Short-chain carboxylic acid PFAS are also used to produce fluorinated organic compounds as a fluoropolymer processing aid. This processing aid improves mixing efficiency between the product and starting materials to maximize synthesis yield.

##### Betaine

Betaine is a zwitterion that facilitates higher aqueous solubility [37]. Thus, betaine-containing PFAS have been used for aqueous film-forming foam. A 6:2 fluorotelomer sulfonamide alkylbetaine (6:2 FTAB) is the most widely used emerging PFAS for aqueous film-forming foam in the second generation (Figure 1). For example, a suspect screening of an aqueous film-forming foam agent identified betaine as a major PFAS constituent [37].

##### Phosphoester

Phosphoester has an amphipathic property with a hydrophilic phosphoester group and several hydrophobic fluorinated chains [38]. This group has been used for food packaging because of its high affinity to paper, high water and oil repulsion properties, and excellent high-temperature stability [38]. 6:2 Fluorotelomer phosphate diester (6:2 diPAP) is a widely used emerging PFAS in this group.

### 2.2. Ether-Type PFAS

Ether-type PFAS contain an ether-linked backbone structure, which is expected to degrade easily under atmospheric conditions [39,40]. These compounds are often used as processing aids for fluorinated compound production, as PFAS alternative materials for this application still appear difficult to develop (Table 2). In terms of substrate and product solubility, carboxylic acids have been empirically considered the functional group of newly generated emerging PFAS.

#### Ethercarboxylic Acid

Ammonium salt of hexafluoropropylene oxide dimer acid (GenX) is a well-known ethercarboxylic acid PFAS produced by Chemours/Dupont (Figure 1) [18]. It was developed as an alternative to PFOA as a fluoropolymer processing aid. Ammonium 4,8-dioxa-3H-perfluorononanoate (ADONA) is another common ethercarboxylated PFAS produced by 3M. It is also used as a fluorotelomer processing aid. ADONA is added at 0.1–1% to the substrate fluorinated monomer to ensure effective mixing. Perfluoro(2-ethoxy-2-fluoroethoxy)-acetic acid (EEA) is a newly developed emerging PFAS used as a fluoropolymer processing aid [16]. Its environmental concentration has been reported using a validated methodology. From a synthetic point of view, GenX was synthesized by dimerization of hexafluoropropylene oxide [16]. ADONA is synthesized via a perfluoroalkylether intermediate by 3M [16]. An oxymethylene was inserted into a fluoroolefin by ultraviolet (UV)-mediated direct insertion, leading to a partially fluorinated ether.

### 2.3. Cyclic PFAS

Perfluoroethylcyclohexylsulfonate (PFECHS) is synthesized by ECF from a cyclohexane- or benzene-containing ethylsulfonate [36]. These cyclic starting materials are fluorinated in hydrogen fluoride by an ECF reaction. This is a mixture of stereoisomers. PFECHS has been developed as a replacement for PFOS. Major applications include anti-erosion in aircraft hydraulic fluid. Additionally, PFECHS was used as a detergent for chrome (VI) plating solution and as an additive or a detergent for photoresist in semiconductor production [36].

### 2.4. Polymers

#### 2.4.1. Fluoropolymers

Fluoropolymers contain fluorine in the main chain of the polymer [49]. Polytetrafluoroethylene (PTFE) has excellent high-temperature stability, high chemical resistance, anti-adhesiveness, and high electrical insulation properties. This is used for packing materials in automobiles and aircraft, chemical plants, and cookware. Fluorinated ionomers are unique fluorinated materials with hydrophobic conductive materials used for proton exchange membranes in fuel battery [50].

#### 2.4.2. SCFP

Side-chain fluorinated polymers contain fluorinated substances in the side chain. Several polymers such as acrylate, urethane, and oxetane have been synthesized [49]. An example includes fluorotelomer-containing polyacrylate, which is used as a coating agent in textiles, food contact materials, and painting materials [51]. For industrial synthesis, a polymerization of side-chain fluorinated monomers is widely used rather than other synthetic methods such as esterification of fluorotelomers to polyacrylate. Thus, this provides a higher esterification ratio in the PFAS-treated products.

### 2.5. Ultrashort-Chain PFAS

**Figure 3:**
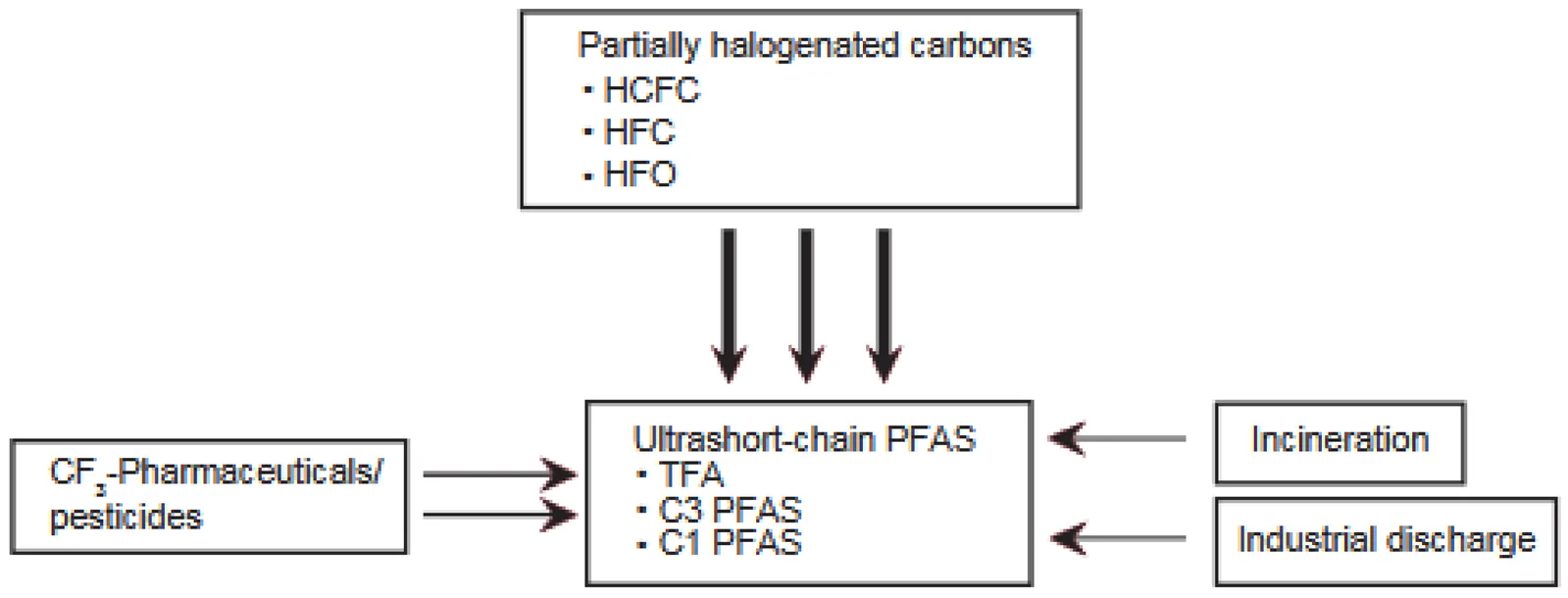
Formation of ultrashort-chain PFAS. Partially fluorinated carbons, CF_3_-pharmaceuticals/pesticides, incineration, and industrial discharge are major pathways for ultrashort-chain PFAS formation. Note that partially fluorinated carbons generate most ultrashort-chain PFAS.

Ultrashort-chain PFAS includes TFMS (C1), TFA (C2), perfluoropropanoic acid (PFPrA, C3), and related C1–C3 compounds, which have attracted much attention in today’s PFAS research [52]. These PFAS might not be produced for industrial purposes but are present in the environment. These PFAS could arise from the following pathways [1] (Figure 3). First, in the presence of a hydroxyl radical in air, partially fluorinated carbons can be converted into trifluoroacetyl fluoride [53]. Its degradation leads to TFA and hydrogen fluoride in the presence of water very rapidly. Ultrashort-chain PFAS are a concern in remote areas, such as the Arctic [54] and Antarctica [54]. Fluorexine is a CF_3_-pharmceticals/pesticides with a selective serotonin reuptake inhibitor property that contains an aryl-CF_3_ whose photochemical decomposition mechanism to TFA has been studied [55].

### 2.6. Ethersulfonate

A mixture of 6:2 Chlorinated polyfluoroalkyl ether sulfonate (6:2 Cl-PFESA) and 8:2 Chlorinated polyfluoroalkyl ether sulfonate (8:2 Cl-PFESA) (F-53B) was first developed in the 1970s [18]. 6:2 Cl-PFESA was synthesized by multi-step reactions consisting of a telomer reaction followed by the introduction of an ether and a sulfonate group [56]. F-53B is a mixture of approximately 85% 6:2 Cl-PFESA and 15% 8:2 Cl-PFESA with associated structural isomers and regioisomers. This is associated with reaction conditions in which the number of taxogen-mediated radical reactions (i.e., *n* = 3 for 6:2 Cl-PFESA and *n* = 4 for 8:2 Cl-PFESA, respectively) may not be precisely controlled. These were used for chromium plating [13].

## 3. Analytical Methods for PFAS Detection

### 3.1. Target Analysis

Mass spectrometry-based techniques are commonly used to quantify PFAS. In particular, regulated compounds such as PFOS and PFOA are quantified using liquid chromatography-tandem mass spectrometry (LC-MS/MS). Because of their historical background, assay procedures for legacy PFAS have been extensively studied [2]. By extending this method, the concentration of the emerging PFAS of interest can be measured. Many protocols have been published for environmental analysis [57]. Specifically, EPA 533 and EPA 537.1 measure PFAS concentrations in drinking water. EPA 533 is a newly developed method that measures 25 compounds, focusing on short-chain PFAS (C4–C12) using weak anion exchange (WAX) solid-phase extraction (SPE) with an isotope dilution technique. EPA537.1 measures 18 long-chain PFAS compounds using styrene-divinylbenzene SPE. Both methods have a detection limit of 2.0 ng/L. EPA8327 and EPA3512 relate to PFAS measurement in non-potable water. EPA8327 describes a method for LC-MS/MS-based quantification of 24 compounds, while EPA3512 presents a method for sample preparation, such as the dilution and filtration of samples. The typical detection limit for this is 10 ng/L. This method is supported by surface water analysis, as this application often finds extraordinarily high PFAS concentrations. For PFAS measurement in animal muscle and plasma, food, and serum, individual protocols for the United States Department of Agriculture (USDA) CLG 2.03 [57], United States Food and Drug Administration (FDA) C-010.02 [58], and Centers for Disease Control and Prevention (CDC) 6304.08 [59] have been established [57]. A current drawback of target analysis is that it fails to quantify the concentration of unknown PFAS whose analytical parameters are not predetermined. Ultrashort-chain PFAS are not included in EPA 537.1, 533, and 1633.

In International Organization for Standardization (ISO) protocols, PFAS concentration must be determined by ISO21675 for both drinking water and non-drinking water, such as wastewater, soil, and biosolids [60]. For food and animal feed, AOAC standard method performance requirements (SMPR) 2023.003 needs to be used [61]. In European protocols, EU2020/2184 [62] is used for drinking water. For other water, the German protocol DIN38407-42 is used in either modified or unmodified forms [63]. For foods and animal feed, the European Union Reference Laboratory (EURL) POPs Guidance has been published.

TFA, TFMS, and PFPrA are well-characterized ultrashort-chain PFAS. Quantifying ultrashort-chain PFAS using LC-MS/MS with an octadecyl (C18) column is challenging because their retention on this analytical column is insufficient. Thus, hydrophilic interaction liquid chromatography or anion exchange chromatography may be used to quantify ultrashort-chain PFAS [30]. For the liquid sample, an optimized liquid–liquid extraction procedure with methyl *tert*-butyl ether (MTBE) was used [64]. In human specimens, TFA is found at detectable concentrations, raising health concerns [65,66,67]. Geologically, TFA has been detected in both industrialized areas and remote areas such as Antarctica [54,68]. In some applications, supercritical fluid chromatography was used [24]. Many extraction techniques have been examined for ultrashort-chain PFAS for gas chromatography-based techniques. For atmospheric samples, solid phase microextraction [69] and passive air sampling [70] were examined.

### 3.2. Suspect Analysis

Suspect analysis semi-quantifies previously annotated PFAS species with high-resolution mass spectrometry. In this case, selected internal standards were used for quantification. As long as the target PFAS has been identified, this method provides an estimation of precursor/derivative concentration. Normally, the database contains several thousand PFAS data; thus, with precise mass values, it is routinely possible to identify PFAS molecular species. Today, some databases are shared and provided by the US EPA and OECD [71].

### 3.3. Non-Target Analysis

Non-target analysis is used for the global monitoring of identified and unidentified PFAS with quadrupole time-of-flight (Q-TOF) mass spectrometry [44,70,72,73,74]. This assay plays a key role in emerging PFAS research. Because fluorinated compounds show characteristic fragmentation patterns, unidentified chemical structures can be estimated in a database-dependent manner. This technique is useful for the identification of unknown compounds using a large set of mass spectral databases. Based on fluorine mass-balance analysis, the major fluorinated compounds in soils are CF_3_-pesticides, followed by TFA [75,76,77,78]. In this context, long-chain and short-chain PFAS are minor components of total fluorinated compounds. In addition to target analysis, this assay detects many other ether-type PFAS, cyclic/aromatic PFAS, and other modified PFAS. Due to aqueous insolubility, quantification of fluoropolymer and side-chain fluorinated polymers is not appropriate by this assay.

### 3.4. Total Oxidizable Precursor (TOP) Assay

TOP assay quantifies both PFAS precursors and target PFAS [71,79]. In order to perform the TOP assay, the samples were aliquoted into two groups. Then, one of them was treated with an oxidant to convert PFAS precursors to perfluorocarboxylic acid (PFCA). By this pretreatment, the precursors for ECF-based perfluorosulfonate (PFSA) and PFCA undergo their original PFSA and PFCA. In FT-based PFAS, this pretreatment converts precursors into multiple PFCAs. In case of 6:2 fluorotelomer sulfonate (6:2 FTS), the decomposition products disturbed at ~23% in C3, ~20% in C4, ~25% in C5, ~17% in C6, and ~5% in C7 [79]. In this assay, the difference between samples with high PFCA and high 6:2 FT-derived PFAS is clear because the former sample contains only a unique PFCA. This assay is performed using LC-MS/MS; thus, it is expected to quantify a small number of samples.

### 3.5. Fluorine Assay

The total fluorine (TF) assay measures total fluorine concentration by combustion ion chromatography without enrichment [80]. Thus, this method can measure the fluorine concentration of legacy and emerging PFAS, including short-chain, ether-type, other PFAS (including cyclic/aromatic), ultrashort-chain, fluoropolymers, and side-chain fluorinated polymers. Extractable organic fluorine (EOF) assay measures activated carbon-extractable fluorine concentration by combustion ion chromatography [81]. Fluorine mass balance (FMB) assay determines the levels of unidentified PFAS by subtracting either TF or EOF from PFAS concentration determined by LC-MS/MS [76,80,82]. Because soil contains CF3-pesticides, FMB typically yields a positive value. This is one reason that the European Union authority has been concerned.

## 4. Degradation and Formation of Emerging PFAS

Current concerns for emerging PFAS share multiple categories that are not deeply involved in legacy PFAS [24,26]. From a chemical and physical point of view, emerging PFAS tend to have a smaller number of carbons, leading to increased volatility. In contrast, the effect of sulfonate and carboxylic acid becomes prominent. This suggests that emerging PFAS have less interaction through hydrophobic interactions. Specifically, emerging PFAS have smaller molecular weights compared to those of legacy PFAS; thus, some emerging PFAS, such as neutral precursor species such as 4:2 fluorotelomer alcohol (4:2 FTOH) and 6:2 fluorotelomer alcohol (6:2 FTOH) have higher volatility. This easily accelerates its evaporation into air, ultimately facilitating long-range atmospheric transport [83]. Second, emerging PFAS show higher water solubility and air diffusion, leading to higher mobility in water in environmental water. Consistent with this property, the removal of these emerging PFAS is less effective by conventional methods such as activated carbons [84]. Third, emerging PFAS are less likely to accumulate in humans. Usually, emerging PFAS are excreted faster than legacy PFAS such as PFOS and longer PFAS [85]. Fourthly, emerging PFAS have been suggested as intermediate contaminants by the decomposition of precursors. For example, partially fluorinated carbons generate PFCAs such as TFA by photooxidation in air. Furthermore, the biological effects of unknown isomers and metabolites have not been unveiled and are a concern [86].

There are three pathways of degradation and formation of emerging PFAS. First, incomplete advanced oxidation processes of PFAS produce emerging PFAS during industrial mitigation. Second, microbial-mediated conversion also leads to a variety of emerging PFAS in CF_3_-pharmaceuticals/pesticides and FT derivatives. Third, photooxidation plays a key role in degrading partially fluorinated carbons in the atmosphere.

### 4.1. Formation of Short-Chain PFAS

#### 4.1.1. Long-Chain/Legacy PFAS as a Starting Material

In this category, an incomplete advanced oxidation process drives the conversion. Specifically, two potential routes generate short-chain PFAS. First, inefficient incineration of long-chain PFAS at low temperature (i.e., 400–500 °C) generates short-chain PFAS [87]. Thus, incineration is currently prohibited in military facilities by law in the United States.

Separately, microbial degradation of legacy PFAS to short-chain PFAS is another pathway for their formation. This increases the accumulation of short-chain PFAS in environmental water due to their higher hydrophilicity. One drawback of bioremediation is that the resulting short-chain fluorinated compounds, such as perfluorobutanoic acid (PFBA) and perfluorobutanesulfonate (PFBS), accumulate in the soil because enzymes no longer react with these substrates. In fact, at neutral pH, PFBS exists as its anionic form. Similarly, most PFBA exists in anionic form, while some exists as the acid form. In sharp contrast, some of their precursors are volatile. The precursors of PFBA are fluorotelomer alcohols such as 4:2 FTOH and 6:2 FTOH, which have volatile properties. The precursors of PFBS are sulfonamides such as *N*-ethylperfluorobutanesulfonamide (EtFBSA) and 2-(*N*-ethylperfluorobutanesulfonamido)ethanol (EtFBSE), these are used for semiconductor production and have volatile properties. Note that ether-containing PFAS, such as GenX and ANODA, are resistant to biotransformation, except under limited conditions [88].

#### 4.1.2. Fluorotelomer PFAS as a Starting Material

In this category, microbial decomposition contributes significantly to degradation. Fluorotelomer-derived PFAS, such as 6:2 FTS, 6:2 diPAP, and 6:2 FTAB, undergo two distinct biotransformation pathways with both aerobic and anaerobic mechanisms [89,90,91]. Under aerobic conditions, the α-carbon of the fluorotelomer PFAS is abstracted by cytochrome P450, followed by oxidation to produce fluorotelomer α,β-unsaturated carboxylic acid (FTUCA) [1]. FTUCA is a unique compound produced from an α-carbon radical due to the strong electronegativity of fluorine. FTUCA then undergoes further degradation, leading to short-chain fluorotelomer carboxylic acid (FTCA). For emerging PFAS, esterases may effectively degrade sulfonamides and phosphoesters, respectively. Usually, anaerobic reactions yield less conversion than aerobic reactions. Note that the oxidation of a double bond by P450 under aerobic conditions normally produces a hydroxylated compound. By contrast, anaerobic reactions produce a variety of reaction products with low yield.

### 4.2. Formation of Ultrashort-Chain PFAS

#### 4.2.1. Short-Chain PFAS as a Starting Material

Essentially, the degradation pathway from short-chain PFAS (C4–C6) to ultrashort-chain PFAS (C1–C3) can be mediated by an incomplete advanced oxidation process through harsh chemical reactions such as electrooxidation, plasma treatment, sonolysis, and other industrial techniques [40,65]. Perfluoroethylsulfonate (PFEtS) is a C2 PFOS analog. Its origin is both short-chain PFOS and the decomposition of sulfonamide to sulfone, as described for PFHxS and other related compounds. Note that enzymes responsible for legacy PFAS fail to react with short-chain PFAS; the contribution of biological processes is negligible.

#### 4.2.2. Partially Fluorinated Carbons as a Starting Material

Photooxidation contributes significantly to the decomposition of partially fluorinated carbons in the air [1,53]. Accumulating evidence has indicated that partially fluorinated carbons, which were not listed in the Montreal Protocol in 1987, can become another source of ultrashort-chain PFAS such as TFA (Figure 3). Specifically, TFA’s precursors include partially fluorinated carbons such as R1234yf, R134a, and R32 [1,53]. In this case, a partially fluorinated carbon species such as R1234yf reacts with a hydroxyl radical to form activated TFA via a peroxyl radical in a gaseous free-radical reaction in the air. Hydrolysis of this TFA precursor gives TFA and hydrogen fluoride. Accumulation of ultrashort-chain PFAS is a concern in remote areas, such as the Arctic [54] and Antarctica [54,68]. This evidence raises concerns that a substantial amount of emerging PFAS seems to be accumulating through previously unanticipated pathways.

#### 4.2.3. CF_3_-Pharmaceuticals/Pesticides as a Starting Material

A combination of microbial degradation and photooxidation plays a major role in mitigating these compounds. CF_3_-Pharmaceuticals/pesticides are emerging PFAS that are distinct from conventional linear PFAS [92]. Their decomposition produces TFA by two separate pathways. First, sunlight-, ultraviolet-, and hydroxyl radical-mediated oxidation of CF_3_-Pharmaceuticals/pesticides generates TFA under aerobic conditions [55]. Second, under aerobic conditions, a variety of hydroxylases and monooxygenases in soil convert these compounds into TFA. This microbial reaction failed to occur under anaerobic conditions when the soil was submerged under water, indicating that anaerobic conditions are not favorable. From a regulatory point of view, European Union authorities and environmental lobby groups argue that TFA should be regulated because of its toxicity to humans [26]. In sharp contrast, the United States EPA considers that CF_3_-pharmaceuticals/pesticides should not be regulated because the chemical reactivity of single fluoro-compounds is dissimilar to that of conventional PFAS [93]. TFA becomes a major PFAS in herbivores and omnivores but not in carnivores [94]. This origin has been postulated as either partially fluorinated carbons or CF_3_-pharmaceuticals/pesticides. In fact, TFA was detected in agricultural products such as wine [95] and fruit [96], accelerating this discussion toward global regulatory action.

## 5. Physicochemical Decomposition of PFAS Mitigation

PFAS are stored around the world in three forms. The first form is stockpiles, especially for aqueous film-forming foam, which was previously produced and is still stored in concentrated form. The second form is disseminated in water, soil, and air. The former is usually concentrated; thus, chemical decomposition works well. The latter specimen shows a low PFAS concentration with a relatively large volume. In some cases, the specimen may be turbid. In these cases, PFAS need to be extracted first, and then the concentrated PFAS are decomposed (Table 3). Overall, physical methods are used to extract PFAS from large volumes of water and soil. Chemical methods destroy PFAS but require substantial energy. Biological methods proceed slowly under mild conditions. This method is occasionally effective when physical and chemical methods are not available. For emerging PFAS, some sulfonate esters fail to bind to WAX resins. Ionic short-chain PFAS are not often retained by hydrophobic interaction. In addition, emerging PFAS are expected to be annihilated rather than just reduced, suggesting that a variety of cost-effective, efficient destruction methods are urgently required (Table 4).

### 5.1. Physical Removal

#### 5.1.1. Activated Carbons

Filtration is a seemingly primitive but widely used technique for PFAS remediation [118]. Many filters can be used based on cost-effectiveness. Biochar is a biologically produced activated carbon with a large surface area that absorbs contaminants such as PFAS. Reverse osmosis is another filtration technique. Although reverse osmosis removes a high volume of PFAS from water, it costs a lot of electricity.

Jiang, T. examined the efficiency of four commercially available adsorbents, namely powdered activated carbon, RemBind 100X, two types of biochar, and two originally synthesized adsorbents, including modified powdered activated carbon and biosorbed aerogel in soil [119]. To do so, each adsorbent was mixed with soil, PFAS was added, and alfalfa was planted for 98 days. Under these conditions, most PFAS, particularly perfluorohexanate (PFHxA), were absorbed by tested adsorbents in the leaf on day 56, rather than in the leaf and root on day 98.

#### 5.1.2. Cyclodextrin Polymers

Cyclodextrin polymers are also a recently developed absorbent for PFAS [120,121]. They effectively absorb short-chain and branched PFAS through host-guest chemistry. To sequester PFAS effectively, cyclodextrin polymers are typically modified with cations such as ammonium groups. These modifiers bind to the head groups of PFAS through ionic interactions. Furthermore, the fluorinated portion of PFAS is captured inside the pores of these polymers through hydrophobic interactions. Cyclodextrin polymer absorbents are recyclable by treatment with ethanol and salt. The efficacy of cyclodextrin polymer has been demonstrated in several lab-scale studies [122]. Based on these preliminary results, Cyclopure, Inc. has commercialized the cyclodextrin polymer absorbent DEXSORB(R).

#### 5.1.3. Metal-Organic Framework (MOF)

MOFs are newly developed adsorbents for PFAS, with high capacity for short-chain PFAS and highly regulated pore sizes [124,125]. This feature is superior to activated carbons and zeolites for selective PFAS binding. A well-studied metal-organic framework comprises zirconium salt and terephthalate [126]. In this case, the zirconium metal center binds to the hydrophobic part of PFAS through electrostatic forces. In contrast, an organic portion of phthalate interacts with the fluorinated chain of PFAS. UiO-66 is a typical Zr-MOF in this area, synthesized from a zirconium salt and terephthalic acid in dimethylformamide. This compound forms a stable special configuration of the cubic crystal system. UiO-67 is another compound synthesized from a zirconium salt and biphenyl-4,4′-benzene dicarboxylate with a much larger pore size compared to UiO-66. Thus, as anticipated, UiO-67 shows higher affinity for PFOS, while both exhibit similarly low affinity for TFA.

## 6. Chemical Removal of PFAS Mitigation

### 6.1. Chemical Removal

#### Ion Exchange

Ion exchange is used to capture ionic substances. Anion exchange resin absorbs PFOS, PFOA, and other negatively charged PFAS [101,102]. Because of its cost-effectiveness, ion exchange resin can be disposable in most cases, such as Purelite A532E (Eco Lab, Horsham, PA, USA). Regenerable products are also available, such as Purelite A520E/A600E and Sorbix RePure (Onterris, Anaheim, CA, USA).

For emerging PFAS, ultrashort-chain PFAS are captured more effectively by ion exchange resin than by activated carbons or other materials that rely on hydrophobic interactions. Current concerns relate to the enrichment of neutral PFAS, such as sulfonamides, which are inappropriate for enrichment. For this reason, ion exchange is combined with other methods, such as activated carbons, to enhance pretreatment yield.

### 6.2. Chemical Decomposition

#### 6.2.1. Incineration

Incineration has been employed for many years in industrial waste material treatment. The current incineration technique for PFAS mitigation involves operating at or above 850 °C for PFOS and 1100 °C for PFOA [42]. A limited number of incineration facilities can accommodate these harsh conditions. The incineration temperature for PFOS is lower than that for PFOA because the bond dissociation energies between C-S and C-C are 72 and 85 kcal/mol, respectively. In both cases, hydrogen fluoride is a major product of this reaction; therefore, neutralization is essential. Low-temperature combustion (400–500 °C) leads to incomplete degradation of short-chain PFAS species, thus causing their atmospheric diffusion. Thus, incineration of waste materials from military facilities has been prohibited in the United States [103].

#### 6.2.2. Supercritical Water Oxidation (SCWO)

SCWO is an extensively studied physicochemical method for PFAS decomposition in the United States [103]. Water becomes supercritical at 218 atm (22.1 MPa), 374 °C. PFAS decomposition proceeds under these harsh conditions. Radical reactions are a major process of PFAS decomposition. In other words, the elimination of CO_2_ or SO_3_ generates a carbon-centered radical, leading to a short-chain fluorinated radical. The decomposition reaction is accelerated in the presence of oxidants, such as hydrogen peroxide and hydroxyl radicals. An application at the Peterson Space Force Base in the United States was reported [106]. Accumulating evidence shows that SCWO is currently applicable to stored aqueous film-forming foam (AFFF)-contaminated water rather than landfill leachate. The differences between these two matrices are both PFAS concentration and matrix complexity. PFAS concentrations in AFFF-contaminated water are 1–2 orders of magnitude higher than in landfill leachate. The matrix of AFFF-contaminated water is much simpler because this water normally contains PFAS as the only contaminants. In sharp contrast, landfill leachate contains ammonium and chloride with a high value of chemical oxygen demand [103,127,128].

#### 6.2.3. Electrooxidation

The electrooxidation technique decomposes PFAS under harsh chemical conditions [102,103]. Essentially, PFAS on the anode in an aqueous solution is oxidized to produce a PFAS-derived radical. In this reaction, an electron on the electrode needs to be transferred to aqueous PFAS, leading to an α-carbon-centered PFAS radical formation. Once produced, this free radical undergoes decarboxylation. Subsequently, this decarboxylated PFAS radical reacts with a short-chain PFAS radical. The boron-doped diamond electrode has been used selectively for this purpose because of its broader potential window [108,138,139]. Direct treatment by electrooxidation may be possible when the water contains less organic contamination or has higher conductivity [140,141]. In contrast, water with high organics, low conductivity, and high chlorine concentration requires pretreatment because the efficiency of electrooxidation on the surface of a boron-doped diamond electrode decreases [129].

#### 6.2.4. Plasma

Plasma is an activated form of electrons and ions in the gaseous phase [103]. Concentrated PFAS can be degraded under these conditions. An activated electron in plasma reacts with PFAS, followed by the formation of a perfluoroalkyl radical. The advantages of plasma for PFAS mitigation include low energy consumption, no chemical additives, reduced treatment time, and almost complete PFAS mitigation [130,131]. However, this method also has drawbacks, such as low efficiency for short-chain PFAS, pH acidification, and formation of short-chain PFAS as a side reaction [132]. More importantly, current technology hampers the scale-up of mitigation apparatus. Recently, methods for atmospheric pressure plasma PFAS decomposition have been developed [109,110,111]. Plasma PFAS decomposition usually requires clean materials. In cases of atmospheric plasma decomposition, therefore, sludge is not acceptable, but algae-filled water may be processed after filtration.

#### 6.2.5. Sonolysis

Sonolysis is a unique method for PFAS decomposition [112]. As PFAS is an amphipathic compound, it accumulates at the interface of bubbles (i.e., between air and water) when a supersonic wave is applied. Therefore, only external energy is applied at this interface at high temperatures, leading to efficient PFAS decomposition.

In reality, because PFAS-contaminated areas require processing enormous amounts of water or soil, no single method meets the economic requirements for PFAS mitigation. Therefore, a combination of at least two methods described above should be performed. Most PFAS can be removed by either filtration or the ion exchange method at low processing cost. A second-tier mitigation method varies depending on availability. SCWO has been implemented in the United States and the European Union, while incineration is common in Japan.

## 7. Biological Remediation

### 7.1. Bioremediation

Bioremediation uses an enzyme for PFAS degradation. Notably, the obligate anaerobe *Acidimicrobium* sp. strain A6 has been extensively studied [134,135]. The reductive dehalogenase A6RdhA enzyme is responsible for PFAS decomposition. This enzyme catalyzes PFAS defluorination at the corrinoid ring of the active center in the presence of ammonium ions or gaseous hydrogen as an electron donor, producing short-chain PFAS. Although this technique seems attractive, PFAS mitigation by this method requires a decade and results in incomplete decomposition. Thus, current research has targeted improving this outcome.

### 7.2. Phytoremediation

Another biology-mediated strategy is phytoremediation, which uses plants to absorb PFAS from soil into plant tissue (Table 5). Before PFAS-targeted remediation, many plant species were tested in the study for other environmentally contaminated materials, such as particulate matter, volatile organic compounds, heavy metals, and greenhouse gases [142]. Based on these results, several species are known to be effective in phytoremediation. This method has several advantages and disadvantages. The main advantage of this technique is that it is suitable for wider field areas. Upon absorption, long-chain PFAS tend to remain in the roots after absorption, while short-chain PFAS easily move to the leaves. Importantly, plants absorb PFAS but do not degrade them in phytoremediation. Thus, secondary mitigation techniques, such as hydrothermal liquefaction, have been developed [100,136,137]. The enhanced effect of PFAS absorption by several adsorbents in soil was examined using Alfalfa as the benchmark [119].

#### 7.2.1. Legumes

The advantage of legume species for phytoremediation is their high nitrogen fixation capacity [119]. This can be achieved through the symbiosis of the host with rhizobia. Therefore, additional nitrogen administration is not always necessary for plant growth in legumes.

##### Alfalfa (*Medicago sativa*)

Alfalfa is a widely distributed plant worldwide. It has a unique characteristic that makes it suitable for phytoremediation [143]. First, alfalfa is resistant to strong stress, thus making it suitable for restoration studies. Second, alfalfa has a large root system, making it highly resistant to heavy winds. A 30-day indoor study demonstrated a high PFAS concentration in the shoot [143]. The bioconcentration factor of PFOS for roots was higher than that for shoots. A translocation factor, defined as the ratio of concentration in shoots to that in roots, was found to be highest in perfluoropentanoic acid (PFPeA) (C5). When this plant was treated with salt as a model for environmental stress, the PFAS concentrations detected in the roots and shoots remained unaltered, suggesting that alfalfa is highly resilient to PFAS accumulation under stress conditions. In a separate experiment, alfalfa effectively absorbed PFOS, PFHxS, and perfluorononanoic acid (PFNA), while GenX was absorbed to a lesser extent [119].

##### Soybean (*Glycine max* (L.) Merr.)

Soybean is a high-protein production plant with a possible high PFAS accumulation capability [144]. PFAS concentrations in soybean planted in an experimental pot with biosolid-applied soil decreased significantly, indicating that soybean is effective for PFAS phytoremediation. Compared with the roots, soybean leaves contain high concentrations of PFBA, while long-chain PFAS fail to accumulate in them. Under these conditions, aquaporin gene expression increased significantly with PFAS concentration, suggesting that water uptake through aquaporins is likely to increase.

#### 7.2.2. Brassica

Brassicaceae has been used for phytoremediation for three reasons [147]. First, it has a high capacity to absorb contaminated metals in soil. Second, it has a larger biomass. Third, it is resistant to glucosinolates, a pungency component of Brassicaceae, for regulating soil microorganisms.

##### Field Mustard (*Brassica rapa*)

Currently, the development of legacy PFAS-free novel compounds for aqueous film-forming foam is in progress. To meet environmental demands, phytotoxicity needs to be determined. Wu et al. reported that *Brassica rapa* can be used as a testing plant for the chronic toxicity of PFAS-free aqueous film-forming foam [145]. In this study, the authors examined seven formulations, including one short-chain PFAS aqueous film-forming foam, four commercial PFAS-free formulations, and two developmental PFAS-free formulations. They proposed that no or low-phytotoxic aqueous film-forming foam would be preferable, as some aqueous film-forming foam showed toxicity in the growth and reproduction of *Brassica rapa*, raising the possibility of damaging forests.

##### Turnip (*Brassica rapa* L. *silvestris*)

Melo et al. examined turnip (*Brassica rapa* L. *silvestris*) to determine the ecotoxicological effect of organoclay on PFAS absorption in soil [147]. In this case, turnips showed reduced shoot biomass, but not root growth, in the presence of organoclay, demonstrating that turnips can be used as a plant model for phytoremediation testing.

#### 7.2.3. High-Biomass Producing Trees

High-biomass-producing trees are fast-growing trees with many leaves and branches and a thick trunk. Würth et al. reported that the amount of absorbed PFAS was in the order of white willow > black poplar > black alder [148]. In this study, PFAS accumulation in the leaves of these trees on PFAS-contaminated soil in Germany was quantified. Short-chain PFAS (C3–C7) predominated at this site.

#### 7.2.4. Weeds and Miscellaneous Plants

##### Hemp (*Cannabis sativa*)

Hemp was used in the phytoremediation study of the former Loring Air Force Base in Maine, the United States [114,136]. Due to the high PFAS concentration associated with aqueous film-forming foam in this area, the air base had already closed. In a preliminary study, it was found that 8:2 fluorotelomer sulfonate (8:2 FTS) was highly detected in the drainage area but low in the berm soil. Based on this evidence, hemp plants were planted for this phytoremediation study in collaboration with the Connecticut Agricultural Experiment Station. As a result, the bioaccumulation factor for PFPeA and PFBA in the leaves was extensively elevated, particularly in areas of high PFAS concentration, demonstrating that phytoextraction was successful.

## 8. Future Perspectives

In conclusion, PFAS are useful chemicals for humans. The replacement of legacy PFAS with emerging PFAS or non-fluorinated materials is progressing successfully. The discovery of PFAS alternative materials in essential areas such as semiconductor production and clinical applications needs to be achieved. Due to a rapid increase in new application areas such as lithium batteries, an efficient recycling procedure needs to be established. Regulatory discussions are expected to reach a practical compromise on risk evaluation for humans, environmental protection, and economic benefits.

## Figures and Tables

**Table 1:** Six major categories of industrial PFAS applications: a generation transition from legacy PFAS to PFAS alternative materials. The required function for particular application was also described.

Category	Function	Generation			Ref.
		Legacy PFAS (mainly long-chain PFAS)	Emerging PFAS	PFAS alternative materials	
Aqueous film-forming foam	Foam stability; self-healing property.	PFOS	6:2 FTAB	Synthetic fluorine-free foam (eg Xanthan gum etc).	[4,5]
Textiles, outdoor goods	Water, oil, and stain repellent; abrasion resistance.	PFOA	6:2 FTSA	Silicone, paraffin, polyurethane.	[7,8]
Food contact materials	Barrier properties against hot oil and water.	8:2 diPAP, PFOA	6:2 diPAP	Cellulose, starch, and plant-derived wax.	[6,9,10,11]
Chromium plating	Surface tension reduction under strong acid and high temperature conditions.	PFOS	F-53B, 6:2 FTS	Not available due to process conversion to chromium(III) plating method.	[12,14]
Semiconductor precision cleaning	Penetration into ultrafine structure; strong acid resistance; high volatility.	PFOS	PFBS, FBSEE	Specialized hydrocarbon surfactant.	[15,19,20,21]
Fluoropolymer processing aid	Maintaining polymerization stability.	PFOA	GenX, ADONA	Non-fluorinated dispersants.	[16,17,18]

**Table 2:** Structure and major chemical characteristics of legacy and emerging PFAS. Emerging PFAS categorized by structural characteristics, functional group, production method, major use, and chemical characterization.

Structural Characteristics	Functional Group	Production Method	Typical Compounds	Major Use and Chemical Characterization	Ref.
Long-chain	Long-chain PFAS (C8–C12)	Sulfonate: 100% (ECF). Carboxylic acid: 20–30% (ECF), 70–80% (FT).	PFOS, PFOA, PFNA, PFDA	General use. Water and oil repellents.	[41]
Long-chain	Chlorofluoroether sulfonate	Telomer reaction followed by an introduction of ether and sulfonate group.	F-53B (6:2 Cl-PFESA + 8:2 Cl-PFESA)	Chromium plating mist suppressants. Widely used as a substitute for PFOS, particularly in China.	[7,13,25]
Short-chain	Sulfonate	ECF	PFBS	Used for aqueous film-forming foam and an additive for photoresist in semiconductor manufacturing.	[42,43]
Short-chain	Sulfonamide	ECF	FBSEE, FBSA	Additives for semiconductor photoresists, water and oil repellents for textiles and paper.	[3,20,37,42,44]
Short-chain	Carboxylic acid	FT	PFHxA	Used as an emulsifier for polymer synthesis such as PTFE and a coating agent for textiles surface.	[43,45]
Short-chain	Phosphoester	FT	6:2 diPAP	Oil-resistant coating for food contact materials.	[46,47,48]
Short-chain	Betaine	FT	6:2 FTAB	Aqueous film-forming foam.	[25,38]
Ether	Ethercarboxylic acid	Dimerization of hexafluoropropylene oxide (GenX). UV-mediated direct insertion of oxymethylene into fluoroolefin (ADONA).	GenX (HFPO-DA), ADONA	Fluoropolymer processing aids for PFTE.	[18,39]

**Table 3:** PFAS remediation methods in use and under investigation. Method was categorized with mode of action, method/devices, major process, and characteristics.

Mode of Action	Category	Method/Devices	Major Process	Characteristics	References
Physical	Absorption	Activated carbons, biochar, organoclay, reverse osmosis, cyclodextrin polymers, metal-organic framework.	Absorption, reverse osmosis membrane.	Efficiently capture low-concentration PFAS in water. Physically blocking PFAS to produce highly purified water.	[74,97,98,99]
Chemical	Ion exchange	Ion exchange resin	Weak anion exchange	Mostly single use.	[100,101,102]
	Incineration	Optimized thermo-decomposition condition	Combustion above 850 °C (PFOS) or 1100 °C (PFOA).	Thermal destruction of PFAS on spent activated carbon at extreme temperature to cleave C-F bonds.	[103,104,105]
	Supercritical water oxidation	Pressure vessel	At 22.1 atm, 374 °C or more harsh condition	Mobile facility has been developed.	[103,106,107]
	Electrooxidation	Boron-doped diamond electrode	Hydrogen peroxide and hydroxyl radical as an oxidant.	Apply powerful radicals and electron transfer mechanisms to decompose PFAS concentrates and short-chain species.	[100,102,103,108]
	Plasma	Gaseous reaction	Oxidation in plasma	Atmospheric pressure plasma decomposition is available. Clear solution would be preferable.	[103,109,110,111]
	Sonolysis	Sonochemical reactor/ultrasonic homogenizer	Electrooxidation of PFAS at the interface of bubble.	PFAS tend to be concentrated at air-water interface due to its surfactant property.	[103,112]
Biological	Bioremediation	Enzyme-mediated degradation	Dehalogenase, P450	Cost-effective and sustainable defluorination of vast groundwater contamination. Both aerobic and anaerobic conditions may be used.	[89,90,91,113]
	Phytoremediation	Absorption in the root and leaf	Plant-mediated absorption	Both annual and perennial plants are used.	[113,114,115,116,117]

**Table 4:** Comparison of physico-chemical/chemical and biological remediation in terms of efficiency, cost-effectiveness, environmental impact, and feasibility.

Chemical/Physical	Removal/Decomposition	Method	Efficiency	Cost-Effectiveness	Environmental Impact	Feasibility	Ref.
Physical	Removal	Activated Carbons	Moderate to Low (short-chain/GenX); high (legacy PFAS)	High	High secondary waste	Very High	[118,119]
Physical	Removal	Cyclodextrin Polymers	High for both long/short-chain and GenX	Moderate	Low	Moderate	[120,121,122,123]
Physical	Removal	Metal-Organic Framework	Very High	Low to Moderate	Low to Moderate	Low	[124,125,126]
Chemical	Removal	Ion Exchange	High for short-chain and GenX (anionic surfactants); better than GAC.	Moderate to High	Moderate	High	[44]
Chemical	Decomposition	Incineration	Very High (>99.9%)	Low	High risk of toxic materials if incomplete.	High	[103]
Chemical	Decomposition	SCWO	Extremely High (>99.99%)	Low to Moderate	Very Low	Moderate	[103,106,127,128]
Chemical	Decomposition	Electrooxidation	High	Moderate	Moderate	Moderate	[129]
Chemical	Decomposition	Plasma	Very High	Moderate	Low	Moderate	[103,109,110,111,130,131,132,133].
Chemical	Decomposition	Sonolysis	High	Low	Low	Low	[103,112]
Biological	Removal	Bioremediation	Low to Moderate	Moderate	Very Low	Moderate	[134,135]
Biological	Removal	Phytoremediation	Low to Moderate	High	Low	High	[100,136,137]

SCWO—Supercritical Water Oxidation.

**Table 5:** Plants selected for PFAS phytoremediation study. Genus/category, scientific name, and common name are presented, along with the plant's advantages.

Genus/Category	Advantage	Scientific Name	Common Name	References
Legume	Through symbiosis with rhizobacteria, it sustains growth in degraded and contaminated areas without fertilizers. The extensive rooting depth enables the remediation of deep-seated contamination.	*Medicago sativa*	Alfalfa	[119,143]
		*Glycine max* (L.) Merr.	Soybean	[144]
Brassicaceae	A fast-growing species characterized by its superior hyperaccumulation potential, enabling the uptake of PFAS contaminants into its plant tissues.	*Brassica rapa*	Field mustard	[145]
		*Brassica juncea*	Mustard	[146]
		*Brassica rapa* L. *silestris*	Turnip	[147]
Poaceae	Its substantial size ensures a high cumulative PFAS uptake per individual plant.	*Avena sativa*	Oat	[147]
High-biomass producing trees	The plant’s extraordinary transpiration capacity drives the rapid uptake of PFAS, effectively acting as a hydraulic barrier to prevent groundwater contamination.	*Populus nigr* L.	Black poplar	[148]
		*Salix alba* L.	White willow	[148]
		*Alnus glutinosa* L.	Black alder	[148]
Weeds, miscellaneous	It grows in almost any environment and has a strong tendency to concentrate PFAS at high levels, particularly in its leaves.	*Cannabis sativa*	Hemp	[114,136,146,149]
		*Helianthus annuus*	Sunflower	[146]

## Data Availability

No new data were created or analyzed in this study. Data sharing is not applicable to this article.

## Abbreviations

4:2 FTOH	4:2 Fluorotelomer alcohol
6:2 Cl-PFESA	6:2 Chlorinated polyfluoroalkyl ether sulfonate; 2-((6-Chloro-1,1,2,2,3,3,4,4,5,5,6,6-dodecafluorohexyl)oxy)-1,1,2,2-tetrafluoroethanesulfonate
6:2 FTAB	6:2 Fluorotelomer sulfonamidoalkyl betaine; Carboxymethyldimethyl-3-[[(3,3,4,4,5,5,6,6,7,7,8,8,8-tridecafluorooctyl)sulfonyl]amino]propylammonium hydroxide
6:2 FTOH	6:2 Fluorotelomer alcohol; 3,3,4,4,5,5,6,6,7,7,8,8,8-Tridecafluorooctan-1-ol
6:2 FTS	6:2 Fluorotelomer sulfonate; 3,3,4,4,5,5,6,6,7,7,8,8,8-Tridecafluorooctane-1-sulfonate
6:2 diPAP	6:2 Fluorotelomer phosphate diester; Bis(3,3,4,4,5,5,6,6,7,7,8,8,8-tridecafluorooctyl) hydrogen phosphate
8:2 Cl-PFESA	8:2 Chlorinated polyfluoroalkyl ether sulfonate; 2-((8-Chloro-1,1,2,2,3,3,4,4,5,5,6,6,7,7,8,8-hexadecafluorooctyl)oxy)-1,1,2,2-tetrafluoroethanesulfonate
8:2 FTOH	8:2 Fluorotelomer alcohol; 3,3,4,4,5,5,6,6,7,7,8,8,9,9,10,10,10-Heptadecafluorodecan-1-ol
8:2 FTS	8:2 Fluorotelomer sulfonate; 3,3,4,4,5,5,6,6,7,7,8,8,9,9,10,10,10-Heptadecafluorodecane-1-sulfonic acid
ADONA	Ammonium 4,8-dioxa-3H-perfluorononanoate; 2,2,3-Trifluoro-3-[1,1,2,2,3,3-hexafluoro-3-(trifluoromethoxy)propoxy]propanoic acid ammonium salt
AFFF	aqueous film-forming foam
C18	Octadecyl
CDC	Centers for Disease Control and Prevention
ECF	Electrochemical fluorination
ECHA	European Chemicals Agency
EEA	Perfluoro(2-ethoxy-2-fluoroethoxy)-acetic acid; 2,2-difluoro-2-[1,1,2,2-tetrafluoro-2-(1,1,2,2,2-pentafluoroethoxy)ethoxy]acetic acid
EOF	Extractable organic fluorine
EPA	Environmental Protection Agency
EURL	European Union Reference Laboratory
F-53B	A mixture of 6:2 Cl-PFESA and 8:2 Cl-PFESA
EtFBSA	*N*-ethylperfluorobutanesulfonamide
EtFBSE	2-(*N*-ethylperfluorobutanesulfonamido)ethanol
FDA	Food and Drug Administration
FMB	Fluorine mass balance
*N*-MeFOSE	*N*-Methylperfluorooctanesulfonamidoethanol
FT	Fluorotelomer
FTCA	Fluorotelomer carboxylic acid
FTUCA	Fluorotelomer α,β-unsaturated carboxylic acid
GenX	Ammonium salt of hexafluoropropylene oxide dimer acid; Ammonium 2,3,3,3-tetrafluoro-2-(heptafluoropropoxy)propanoate
ISO	International Organization for Standardization
LC-MS/MS	Liquid chromatography-tandem mass spectrometry
MTBE	Methyl *tert*-butyl ether
MOF	Metal-Organic Framework
OECD	Organization for Economic Cooperation and Development
PFAA	Perfluoroalkyl acid
PFAS	Per- and polyfluoroalkyl substances
PFBA	Perfluorobutyric acid
PFBS	Perfluorobutanesulfonate
PFCA	perfluorocarboxylic acid
PFECHS	Perfluoroethylcyclohexylsulfonate
PFEtS	Perfluoroethylsulfonate
PFHxA	Perfluorohexanate
PFHxS	Perfluorohexanesulfonate
PFNA	Perfluorononanoic acid
PFOA	Perfluorooctanoic acid
PFOS	Perfluorooctanesulfonate
PFOSA	Perfluorooctanesulfonamide
PFPeA	Perfluoropentanoic acid
PFPrA	Perfluoropropionic acid
PFSA	perfluorosulfonate
PTFE	Polytetrafluoroethylene
Q-TOF	quadrupole time-of-flight
SAmPAP	Perfluorooctane sulfonamidoethanol-based phosphate
SCFP	side-chain fluorinated polymers
SCWO	Supercritical water oxidation
SMPR	standard method performance requirements
SPE	Solid phase extraction
TF	Total fluorine
TFA	Trifluoroacetic acid
TFMS	Trifluoromethanesulfonate
TOP	Total oxidizable precursor
USDA	United States Department of Agriculture
WAX	Weak anion exchange
